# Dietary Protection against Cognitive Impairment, Neuroinflammation and Oxidative Stress in Alzheimer’s Disease Animal Models of Lipopolysaccharide-Induced Inflammation

**DOI:** 10.3390/ijms24065921

**Published:** 2023-03-21

**Authors:** Davide Decandia, Francesca Gelfo, Eugenia Landolfo, Francesca Balsamo, Laura Petrosini, Debora Cutuli

**Affiliations:** 1 IRCCS Fondazione Santa Lucia, Via Ardeatina 306, 00179 Rome, Italy; 2 Department of Psychology, Sapienza University of Rome, Via dei Marsi 78, 00185 Rome, Italy; 3 Department of Human Sciences, Guglielmo Marconi University, Via Plinio 44, 00193 Rome, Italy

**Keywords:** Alzheimer’s disease, cognition, neuroinflammation, oxidative stress, neurodegeneration, animal models

## Abstract

Alzheimer’s disease (AD) is a rapidly growing epidemic with a heavy social and economic burden. Evidence suggests that systemic inflammation, dysregulation of the immune response and the resulting neuroinflammation and neurodegeneration play a significant role in AD pathogenesis. Currently, given that there is no fully convincing cure for AD, the interest in lifestyle factors (such as diet), which potentially delay onset and reduce the severity of symptoms, is increasing. This review is aimed at summarizing the effects of dietary supplementation on cognitive decline, neuroinflammation and oxidative stress in AD-like animal models with a focus on neuroinflammation induced by lipopolysaccharide (LPS) injection, which mimics systemic inflammation in animals. The compounds reviewed include curcumin, krill oil, chicoric acid, plasmalogens, lycopene, tryptophan-related dipeptides, hesperetin and selenium peptides. Despite the heterogeneity of these compounds, there is a strong consensus on their counteracting action on LPS-induced cognitive deficits and neuroinflammatory responses in rodents by modulating cell-signaling processes, such as the NF-κB pathway. Overall, dietary interventions could represent an important resource to oppose AD due to their influence in neuroprotection and immune regulation.

## 1. Introduction

The constant and rapid growth in the proportion of older people worldwide and the increased incidence of cognitive decline and dementia have strong impacts on society. In fact, the burden of late-life neurodegenerative diseases does not involve only patients but also their families and the healthcare system. The incidence of dementia rises with age [1]. As stated by the World Health Organization (WHO), the number of people suffering from dementia worldwide is likely to triple by 2050, and Alzheimer’s disease (AD) is one of the main causes of dementia [2]. In AD, extracellular amyloid-β (Aβ) deposits and intracellular phosphorylated Tau aggregates form senile plaques and neurofibrillary tangles, respectively, two neuropathological hallmarks that induce neuroinflammation and subsequent neuronal damage associated with cognitive decline and neuropsychiatric symptoms [3,4,5,6,7].

Neuroinflammation accompanies central nervous system (CNS) injury, infection, toxicity and autoimmunity [8]. When transiently activated, inflammatory responses involve the release of cytokines and growth factors favorably affecting the post-injury tissue. On the other hand, chronic or uncontrolled inflammatory responses may lead to detrimental processes linked to the pathological progression of neurodegenerative disorders, such as dementia, Parkinson’s disease, multiple sclerosis and amyotrophic lateral sclerosis [9,10]. 

Notably, several studies have indicated that neuroinflammation plays a critical role in the AD pathogenesis [8,11]. Neuroinflammation is expressed by the activation of microglial cells and astrocytes, which release and respond to a wide variety of chemokines and pro-inflammatory cytokines (e.g., tumor necrosis factor α (TNF-α) and interleukin (IL)-1β and IL-6) mediating innate immune responses as well as by the generation of reactive oxygen species (ROS) boosting oxidative stress [12,13,14]. Hence, the treatment of neuroinflammation and oxidative stress appears to be of primary importance in alleviating AD pathology. 

Different agents, such as lipopolysaccharide (LPS), are used to model neuroinflammation, which may trigger and perpetuate neurodegeneration. In fact, neuronal death may induce an inflammatory process that, by itself, may lead to cell death [15]. LPS is one of the major components of the membrane of Gram-negative bacteria [16] and is mostly used to stimulate glial cells, mainly microglia, since it activates various intracellular molecules that alter the expression of a plethora of inflammatory mediators [17,18,19]. Therefore, LPS is commonly used in rodents to elicit neuroinflammation (e.g., to increase pro-inflammatory cytokines) [20] and provoke cognitive dysfunction and brain amyloidogenesis, mimicking AD symptoms [21,22,23]. 

Growing evidence indicates that environmental factors, such as an unbalanced diet and physical inactivity, play an important role in AD development [24]. It is well-known that the diet modulates the immune system and that different nutrients and bioactive components can influence neuroinflammation [25]. For example, polyphenols, unsaturated fats and vitamins A, C and E inhibit oxidative stress and neuroinflammation [26,27], while saturated fats promote neuroinflammation, particularly in the hypothalamus [28].

On the basis of the above, the present narrative review is aimed at accounting for the potential of specific dietary compounds to counteract cognitive decline and neuroinflammatory and/or oxidative stress correlates as observed in AD-like animal models of LPS-induced neuroinflammation. 

## 2. LPS-Induced Neuroinflammation as an Animal Model of Alzheimer’s Disease

LPS is a highly acylated saccharolipid located on the outer leaflet of the outer membrane of Gram-negative bacteria [16,29]. LPS up-regulates several inflammatory mediators, such as inducible nitric oxide synthase (iNOS), cyclooxygenase-2 (COX-2) and pro-inflammatory cytokines, including IL-1β and TNF-α [18,19]. 

The Toll-like receptor (TLR) is a pattern recognition receptor, which plays a central role in the first steps of the immune response—in particular in detecting microbial components in the CNS [30]. The main target of LPS is TLR4. In the brain, TLR4 is expressed in astrocytes, microglia, macrophages and neurons, and its signaling pathway is implicated in eliminating bacteria but can also have a deleterious effect as occurring in the case of Aβ [17]. In fact, similarly to LPS, Aβ oligomers, which are primarily implicated in the pathophysiology of AD, also activate microglia via TLR4. This leads to a chronic release of pro-inflammatory cytokines, which cause neuronal damage and subsequent cognitive impairment [31,32]. LPS is often used in preclinical studies to mimic the condition of increased neuroinflammation featuring neurodegenerative diseases. 

Its use contributes to a better understanding of the intricate relationship between neuroinflammation and AD progression, mainly regarding Aβ processing and deposition. LPS is used in a variety of in vitro (to stimulate cell cultures) and in vivo protocols. In the latter case, it is injected either in the CNS or in the periphery by single or multiple injections. Thus, LPS effects may vary according to the experimental protocols [17]. 

**Figure 1:**
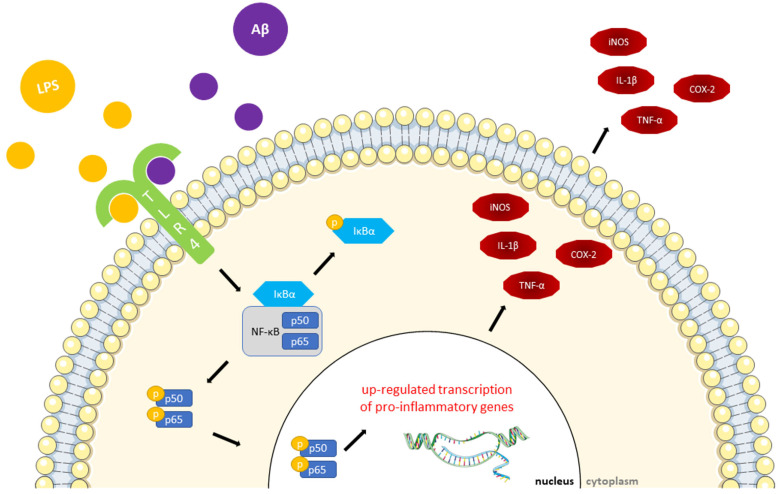
Schematic representation of the LPS-induced inflammatory mechanism mediated by the NF-κB pathway. TLR4 senses LPS and Aβ and triggers the activation of NF-κB. Phosphorylated IκBα permits the NF-κB subunits p50-p65 to translocate into the nucleus, up-regulating the transcription of pro-inflammatory genes and provoking the release of several inflammatory mediators, such as iNOS, COX-2, IL-1β and TNF-α. Parts of the figure were drawn by using pictures from Servier Medical Art. Servier Medical Art by Servier is licensed under a Creative Commons Attribution 3.0 Unported License (https://creativecommons.org/licenses/by/3.0/ accessed on 20 February 2023).

Numerous papers report that intraperitoneal (i.p.) injections of LPS impair learning and memory and boost Aβ production activating microglia, which, in turn, stimulate the nuclear factor kappa B (NF-κB) pathway [33,34]. NF-κB, triggered by TLR4, is one of the key activators of the immune response in the CNS regulating the production of many cytotoxic factors (e.g., iNOS, COX-2, IL-1β and TNF-α) [35] (Figure 1). In addition to causing neuroinflammation, LPS increases caspase activation and intensifies oxidative stress in the brain. This is achieved by stimulating the production of an enormous amount of ROS, which leads to neuronal apoptosis and cognitive impairment. These effects are commonly found in neurodegenerative diseases, including AD [14,36,37,38].

## 3. Impact of Dietary Interventions on the Cognitive and Biochemical Dysregulation Induced by LPS Administration

The interest in diet as a lifestyle factor whose changes could potentially delay the onset and severity of age-related cognitive deterioration is in constant growth. Currently, unhealthy lifestyle is considered to be a risk factor for several pathological conditions, including neurodegenerative diseases, such as AD [24,39]. In the last decade, a wide range of clinical trials showed that appropriate dietary supplementation can lead to numerous beneficial effects both in elderly healthy individuals as well as in AD patients [40,41,42,43,44,45].

The next paragraphs account for the behavioral aspects and molecular mechanisms involved in how different nutrients and dietary components may exert a protective action against cognitive decline, neuroinflammation and oxidative stress produced by the LPS administration used to model AD in rodents. To analyze this topic on the basis of the available evidence, the selection of literature in PubMed included only experimental research studies obtained by searching for the combination of the nutrients/nutraceuticals/dietary enrichment or supplementation/diet, AD, LPS and rodents (mice or rats) keywords. Moreover, we selected the articles in which a cognitive assessment of the animals together with neuroinflammatory and/or oxidative stress correlates were provided. The articles had to be peer-reviewed and written in English without any time frame restriction (up to 31 January 2023). 

We discuss the results obtained in the nine articles that met the selection criteria presented in chronological order (per nutrient). The dietary compounds that emerged from our research are curcumin, krill oil, chicoric acid, plasmalogens, lycopene, tryptophan-related dipeptides (TD), hesperetin and selenium peptides (Se-Ps). The articles taken into consideration for the drafting of this literature review are briefly summarized in Table 1, which provides the methodological specifications for each study (i.e., animal age and strain, kind of dietary supplementation, LPS administration procedure, cognitive assessment methods and biological correlates investigated). In the following, cognitive and behavioral outcomes resulting from the administration of different nutrients to LPS-injected rodents are described in association with anatomical correlates, if present. In a different section, the effects of dietary supplementation on LPS-induced neuroinflammation and oxidative stress are presented.

### 3.1. Dietary Interventions Counteract Cognitive Decline Induced by LPS Administration

The selected studies assessed the effects of the dietary supplementation against LPS-induced cognitive deficits by using the following five validated rodent behavioral tests: Morris Water Maze (MWM), Fear Conditioning (FC) test, step-through/down Passive Avoidance (PA), spontaneous alternation Y-Maze (Y-maze) and Novel Object Recognition Test (NORT) (Table 1). All these tasks model different aspects of the cognition (such as reference memory, working memory and episodic memory) and brain integrity disrupted by AD [55].

MWM is designed to test hippocampal-dependent spatial learning and reference memory in rodents, mainly by measuring their escape latency (or distance travelled) to find a submerged platform during the training phase (spatial learning) and the time spent (or distance travelled) in the target quadrant during the probe phase, when the platform is removed by the water tank (spatial reference memory) [56,57].

FC is an associative learning task in which rodents learn to associate a particular neutral stimulus (e.g., a tone, a scent or a context) with an aversive stimulus (such as a mild electrical foot-shock) showing a conditioned fear response (i.e., freezing) after repeated pairings of neutral and aversive stimuli [58]. The main brain areas involved in cued and contextual FC include the amygdala, hippocampus, frontal cortex and cingulate cortex [59,60].

PA is a test used to assess memory function in which rodents learn to avoid entering an environment (e.g., the dark compartment of the testing chamber) where they had previously been exposed to an aversive stimulus (such as a mild foot-shock), and PA acquisition entails the amygdala, infralimbic prefrontal cortex and fronto-striatal circuits [61].

Y-maze is a test used to measure spatial working memory. In fact, based on their willingness to explore new environments, rodents typically prefer to investigate a new arm of the maze rather than returning to a recently visited one, thus, spontaneously alternating the entered arm [62]. Many brain areas (such as the hippocampus, septum, basal forebrain and prefrontal cortex) are involved in the spontaneous alternation behavior assessed by Y-maze [63].

NORT evaluates recognition memory in rodents based on their natural tendency to spend more time exploring a novel object than a familiar one and is strictly dependent on hippocampal integrity [64,65,66,67].

Interestingly, the studies selected in the present review showed that all the dietary compounds investigated exert beneficial effects against LPS-induced cognitive decline in adult mice and rats.

#### 3.1.1. Dietary Interventions with Curcumin

Curcumin is the active ingredient in the dietary spice turmeric (*Curcuma longa*) and has a wide range of beneficial properties, such as anti-inflammatory, antioxidant, chemopreventive and chemotherapeutic activities, that—combined with non-toxicity—contribute to its promising therapeutic potential [68,69,70]. According to JECFA (Joint United Nations and World Health Organization Expert Committee on Food Additives) and EFSA (European Food Safety Authority) reports, curcumin is totally safe within the Acceptable Daily Intake (ADI) of 0–3 mg/kg [70]. 

Despite the clinical safety, some side effects have been reported in the literature. In fact, individuals receiving a dosage between 0.45 to 3.6 g/day of curcumin for one to four months experienced nausea and diarrhea, accompanied with an increase of alkaline phosphatase and lactate dehydrogenase in the serum [71]. Moreover, a supplementation with curcumin of 500–12,000 mg caused minimal toxicity not related to dosage (diarrhea, headache, rash and yellow stool) in the 30% of healthy subjects [72].

In the study by Kawamoto and colleagues [46], 4 days of supplementation with curcumin conferred neuroprotection against acute neuroinflammation induced by a single LPS i.p. injection in mice. Spatial learning and memory retention were assessed with a MWM protocol envisaging two probe phases—at 4 h and 24 h after the last training session. LPS treatment and curcumin preventive supplementation appeared not to affect short-term memory retention in the 4 h probe phase. Differently, curcumin was able to protect against the LPS-induced impairment of long-term memory consolidation in the 24 h probe phase. In the same animals, Kawamoto et al. [46] investigated contextual and cued memory through the FC test. They found that LPS and curcumin did not affect the amygdala-dependent cued test; however, in line with the MWM results, curcumin pretreatment ameliorated long-term memory retention impairment induced by LPS in the hippocampal-dependent contextual FC paradigm.

It has been described how agonists of TNF receptor (TNFR)-2 ameliorate cognitive functions and AD neuropathology [73,74] while TNFR-1 mediates more deleterious effects on cognition and is up-regulated in AD patients [75]. Interestingly, Kawamoto et al. [46] showed that curcumin was not able to counteract LPS-induced cognitive impairment, sickness behavior and anxiety in TNFR-1 and TNFR-2 double knockout mice, thus, suggesting that curcumin protection against cognitive deficits requires TNFR2 activation.

In another study, Hajipour et al. [47] administered curcumin for 14 days to male rats before a single LPS i.p. injection in order to protect from cognitive decline and neuroinflammation. Curcumin was effective in protecting from memory impairment in the PA test. Furthermore, the curcumin supplementation prevented neuronal loss in the hippocampal cornu ammonis 1 (CA1) and dentate gyrus (DG) and recovered DG long-term potentiation (LTP) impairment induced by LPS [47].

While curcumin ameliorates cognitive decline and synaptic functions in LPS-treated animals [46,47] in agreement with the findings obtained in both in vivo and in vitro other models of AD [76], to date, clinical studies on elderly individuals and patients with AD are still few or ongoing and indicate that curcumin is more effective in improving cognitive function in the elderly than in improving symptoms of AD [77,78].

#### 3.1.2. Dietary Interventions with Krill Oil

In recent years, among nutraceuticals, omega-3 fatty acids have been extensively studied in both physiological and pathological aging conditions [79]. Krill oil, which is extracted from small Antarctic crustaceans, is a supplement that contains the omega-3 fatty acids eicosapentaenoic acid (EPA) and docosahexaenoic acid (DHA). The 2015–2020 Dietary Guidelines for Americans recommended an ADI of approximately 250 mg of EPA and DHA for the general population [80]. 

The EFSA stated that supplemental intakes of DHA alone up to about 1 g/day do not raise any concerns for the general population with a maximum intake of 4–5 g/day of combined EPA and DHA, after which, side effects, including bleeding episodes, impaired immune function, increased lipid peroxidation and impaired lipid and glucose metabolism, can occur [81]. EPA and DHA supplementation is associated with health benefits, including the improvement of cognition, systemic inflammation, lipid metabolism and depression symptoms, given its anti-inflammatory and antioxidant properties [82,83].

In 2017, Choi et al. fed mice with rodent chow supplemented with Antarctic krill oil for four weeks to counteract the adverse effect of 7-day LPS i.p. administration (occurring in the last seven days of supplementation) [48]. In the MWM, LPS-injected mice showed learning deficits consisting in greater escape latency and longer escape distance compared to control mice. On the contrary, krill oil-treated mice injected with LPS displayed improved learning ability and spatial memory in comparison to LPS-injected mice without any supplementation [48]. Moreover, krill oil-treated mice injected with LPS showed increased step-through latency in the PA in comparison to LPS-treated mice [48].

In a similar study, krill oil administration improved memory functions in Aβ25-35-injected mice and concomitantly attenuated neuronal oxidative stress and neuronal apoptosis [84]. Moreover, a randomized controlled trial in healthy elderly individuals reported beneficial effects of a 12-week treatment with krill oil on cognitive functions [85]. No studies on the effects of krill oil are currently available for MCI and AD patients.

#### 3.1.3. Dietary Interventions with Chicoric Acid

Chicoric acid is a caffeic acid derivative that can be found extensively in echinacea (also known as purple coneflower; *Echinacea purpurea*), chicory, lettuce, dandelion and other edible plants and vegetables [86]. It is a nutraceutical with powerful antioxidant, anti-Human Immunodeficiency Virus (HIV), anti-inflammatory and anti-obesity properties [87]. A recent review of the literature regarding the bioactive effect of chicoric acid reported no overdose side effects and no contraindications nor drug interactions [86].

Liu et al. chronically administered chicoric acid to mice that underwent LPS i.p. injections for 9 consecutive days [49]. Chicoric acid prevented LPS-induced working memory deficits in the Y-Maze as well as spatial learning and memory deficits in the MWM [49]. Notably, the cognitive function enhancement obtained by chicoric acid supplementation was supported by a concomitant reduction in LPS-induced hippocampal neurogenesis loss [49].

Furthermore, no study described the effects of chicoric acid supplementation in other animal models of AD. Unfortunately, the current scientific literature is lacking in studies that investigate the potential beneficial effects of a dietary intervention with chicoric acid in the elderly as well as in MCI and AD patients.

#### 3.1.4. Dietary Interventions with Plasmalogens

Plasmalogens are a subclass of cell membrane glycerophospholipids that are widely distributed within the mammalian organism, particularly in the brain. Ascidians, mussels and scallops are foods rich in plasmalogens [88]. Plasmalogens may trigger either an anti- or pro-inflammation response, and importantly they are found to decrease in neurodegenerative and metabolic disorders as well as in aging [89]. In a clinical trial, plasmalogens (1 mg/kg) were administered to MCI and mild AD patients, and no differences in adverse effects were reported between the placebo and treated groups [90].

In a study published in 2018 by Hossain and colleagues, a dietary supplementation with plasmalogens was preventively administered for three months to mice whose cognitive decline was elicited by LPS i.p. injections for 7 consecutive days [50]. Plasmalogens were able to rescue spatial learning and memory retrieval impairment induced by chronic LPS injections in MWM [50]. Similarly, in a recent study using a mouse model of AD with chronic cerebral hypoperfusion, scallop-derived plasmalogens were able to recover cerebral blood flow and reduce cognitive deficits, Aβ, neuroinflammation, oxidative stress and neuronal loss [91]. These findings are in line with the positive effects on cognitive functions found in mild AD patients orally treated with plasmalogens [90]. In general, plasmalogens display promising effects in reducing neuroinflammation and boosting cognitive functions [92].

#### 3.1.5. Dietary Interventions with Lycopene

Lycopene is a red plant pigment found in tomatoes, watermelons, apricots, etc., that has health-beneficial effects, such as antioxidant, anti-obesity and anti-cancer effects [93]. Lycopene has been found to be decreased in MCI and AD patients [94]. In two reports by the WHO and the EFSA, it was stated that lycopene consumption is generally safe within the established ADI of 0–0.5 mg/kg [95,96].

Wang et al. [51] administered long-term (5 week) supplementation with lycopene to mice chronically injected with LPS in the last 9 days of supplementation. Interestingly, lycopene antagonized LPS-induced deleterious effects on working memory in the Y-Maze and on spatial learning and memory in the MWM [51]. 

Other preclinical studies have found that lycopene administration was able to enhance cognitive abilities and neurogenesis counteracting Aβ neurotoxicity, neuroinflammation and oxidative damage in rat models of AD subjected to an intracerebroventricular (i.c.v.) injection of Aβ_1–42_ [97,98,99,100]. These findings are in line with the still-limited evidence from human intervention trials suggesting that increased lycopene intake may enhance cognitive performances in the elderly [84].

#### 3.1.6. Dietary Interventions with Tryptophan-Related Dipeptides (TD)

Several studies highlighted the effects of fermented dairy products on cognitive function as preventive against dementia, including AD [101]. Epidemiological studies found that dairy products could promote healthy brain function during aging [102] and that higher consumption of low-fat dairy products once a week correlated with better cognitive function (such as memory recall) [103]. Apart from allergies and intolerances, fermented dairy products are known to be safe for humans.

Ano [52] and colleagues evaluated the effectiveness of fermented dairy products in contrasting LPS-induced cognitive decline. To this aim, the researchers screened different TD obtained from enzyme-digested milk protein and supplemented mice with them at different concentrations for 8 days before a single i.c.v. injection of LPS. Only the high dosage (30 mg/kg) of tryptophan-tyrosine peptides ameliorated LPS-induced impairment in spatial working memory (observed in the Y-Maze) and recognition memory (assessed by the NORT) [52]. 

Cognitive enhancement promoted by TD supplementation was accompanied by the attenuation of LPS-induced atrophy of the dendritic spines of pyramidal neurons in the hippocampal subregion CA1, which is reported to be crucial for spatial memory and object recognition memory [52,64,66,67]. Using the same dipeptides, Ano and colleagues performed the same supplementation in aged mice and in a transgenic mouse model of AD with 5xFAD mice [52]. In the same study, they found that TD were even able to prevent cognitive decline, microglial alteration and hippocampal LTP deficits in aged mice, while in 5xFAD mice, TD were able to protect from cognitive decline, not only reducing microglial inflammation but also suppressing Aβ accumulation [52].

#### 3.1.7. Dietary Interventions with Hesperetin

Hesperetin is a polyphenolic plant compound from the flavonoid group, derived from hesperidin and predominantly found in citrus fruits, such as oranges and grapefruit, whose intake is associated with a lower risk of neurodegenerative and cardiovascular diseases, along with neuroprotective effects in different models of neurodegenerative disorders [104]. The EFSA reported several studies that investigated the safety of hesperidin in rats, concluding that 500 mg/kg was the maximum dose showing no adverse effects, while the highest dose tested (1000 mg/kg/day) elicited significant alterations in body and organ weight, hematology, clinical chemistry and tissue histopathology [105].

Muhammad et al. supplemented mice with hesperetin for five weeks to prevent the cognitive decline induced by chronic LPS injections in the last two weeks [53]. Hesperetin successfully improved spatial learning and memory, which were strongly damaged by repeated LPS injections. In fact, LPS-treated mice supplemented with hesperetin showed decreased mean escape latency and increased time spent in the target quadrant in the MWM as well as increased spontaneous alternations in the Y-Maze compared to the LPS alone-treated mice [53]. Interestingly, the cognitive improvement induced by hesperetin could result from the nutraceutical counteraction of the detrimental LPS effect on synaptic plasticity. Hesperetin promoted neuronal health and synaptic integrity by enhancing the levels of phosphorylated-cAMP response element binding protein (p-CREB), postsynaptic density protein-95 (PSD-95) and syntaxin [53].

Other preclinical studies indicated the beneficial effects of hesperetin. In aged rats, hesperetin supplementation ameliorated the impairment of emotional memory and hippocampal LTP [106]. In a mouse AD model of Aβ_1–42_ i.c.v. injection, hesperetin enhanced cognition by inhibiting oxidative stress, neuroinflammation and apoptotic cell death [107]. Furthermore, in Aβ_1–42_ i.c.v. injected rats, hesperetin improved memory retrieval and recognition memory consolidation by regulating oxidative stress in the hippocampus [108].

To date there are no clinical trials investigating the effects of dietary supplementation with hesperetin in older adults and in MCI and AD patients.

#### 3.1.8. Dietary Interventions with Selenium Supplements (Se-Ps)

Se-Ps are selenium supplements and good sources of peptides, whose anti-inflammatory and antioxidant effects have the potential to prevent or treat the neuroinflammation related to neurodegenerative disorders, such as AD [109,110]. Edible mushrooms are rich in selenium [111]. An EFSA report stated the adequate intake of selenium, which varies at different ages, as an amount of 70 μg/day for adults and pregnant women. However, it was reported that the maximum amount for selenium intake with no adverse effects ranged between about 200 and 500 μg/day, while higher dosages (850–1000 μg/day) lead to clinical selenosis, a toxicological state characterized by headache, loss of hair, deformation and loss of nails, skin rash, malodorous (garlic) breath and skin, excessive tooth decay and discoloration as well as numbness, paralysis and hemiplegia [112].

Recently, Wu et al. supplemented mice with Se-Ps for 28 days to prevent the cognitive decline elicited by chronic LPS administration in the last 9 days of supplementation [54]. Using the step-down PA test, the Authors found that Se-Ps effectively protect learning and memory capacity impaired by LPS [54]. This result was in line with the beneficial effects of the selenium supplementation in other animal models of AD. In a study using an AD-like rat model of streptozotocin-induced sporadic dementia, an organoselenium compound exerted a therapeutic effect reverting memory impairment and counteracting oxidative stress [113]. 

In a 3xTG mouse AD model, a 3-month supplementation with selenium-enriched yeast ameliorated spatial learning and memory retention, enhanced neuronal activity and decreased the activation of astrocytes and microglia, the synaptic deficits and Tau levels [114]. In a different study using the same mouse model of AD, a chronic 4-month dietary administration of sodium selenate counteracted learning and memory deficits and reduced the number of aggregated Tau-positive neurons and astrogliosis in the hippocampus, leaving the Aβ levels unchanged [115].

Regarding human studies, a positive correlation between cognitive function and selenium serum levels was found in AD patients [116]. One study investigated the potential preventive effect of a dietary supplementation with selenium (alone or in combination with vitamin E) against cognitive decline in older men; however, unfortunately, no effect was found [117].

### 3.2. Dietary Modulation of Neuroinflammatory and Oxidative Stress Biomarkers Up-Regulated by LPS Administration

When considering the impact of dietary bioactives on AD pathophysiology and inflammation, it is mandatory to consider whether and how nutrients could affect AD neuropathological hallmarks, such as Aβ levels. Several studies reported that LPS may increase the brain Aβ burden [17,48,49,50,51]. LPS injections also up-regulate the expression of Aβ precursor protein (APP) and its transmembrane 99-residue C-terminal fragment (C-99) [48,49,51] as well as the levels and activity of β-secretase (BACE1), an enzyme producing Aβ [48,49,118]. Dietary modulation through different compounds is effective in counteracting LPS-induced Aβ accumulation due to neuroprotective action. In fact, Antarctic krill oil, chicoric acid and lycopene were able to decrease the levels of Aβ_1–42_ and down-regulate APP [48,49,51]. Plasmalogen supplementation also diminished the Aβ burden [50]. Furthermore, Antarctic krill oil was able to reduce BACE1, C-99 and β-secretase activity [48]. Chicoric acid reduced the expression of BACE1 [49].

Astrogliosis is a graduated process in which astrocytes undergo different morphological and functional changes in response to brain diseases, including AD [119]. Astrogliosis is characterized by cellular hypertrophy and up-regulation of the cytoskeletal protein glial fibrillary acidic protein (GFAP), often used as a biomarker of astrocytes [120]. As for the immune response in AD, it is important to consider the Aβ-triggered changes in microglial phenotype and functionality that contribute to progression of neuropathological conditions. These changes include altered microglial morphology, impaired Aβ phagocytosis as well as increased neuroinflammatory response, synapse engulfment, neuronal phagoptosis and Tau aggregation [11]. Ionized calcium-binding adapter molecule 1 (IBA-1) is a commonly used marker for activated microglia as displayed in AD [121]. In mice, LPS treatment increased GFAP and IBA-1 levels in the hippocampus and cortex [48,49,50,51,53]. Some nutrients seem efficacious in counteracting this effect. In fact, dietary supplementation with Antarctic krill oil, chicoric acid, plasmalogens or hesperetin was able to down-regulate the expression of both GFAP and IBA-1 in the hippocampus [48,49,50,53] and in the cortex [49,50]. These results are in line with previous studies reporting that omega-3 fatty acids (as EPA and DHA) can cross the blood–brain barrier and promote an anti-inflammatory effect by reducing microglia activation [122]. Moreover, plasmalogens, as they are enriched in lipid rafts [123], could block the internalization of TLR4 and the subsequent activation of this receptor (stimulated by LPS) that is known to induce neuroinflammation [124]. Alternatively, they could inhibit the LPS-induced increased release of inflammatory cytokines in the brain and reduce the overactivation of glia, possibly by modulating the distribution of cytokine receptors onto lipid raft domains [50].

Moreover, in mice, hippocampal GFAP was reduced with curcumin supplementation [46], and IBA-1 was decreased with lycopene supplementation in both the hippocampus and cortex [51].

In AD, the large number of Aβ aggregates and their inhibited phagocytosis are associated with an unbalanced activation of microglia and astrocytes. In fact, activated microglia produce inflammatory cytokines contributing to astrocyte activation (reactive A1 astrocytes with neurotoxic properties), and, in turn, activated astrocytes modulate microglial activation leading to Aβ load increase. The chronic perturbation in the crosstalk among microglia, astrocytes and neurons consequently affects neuronal health and cognitive functions [8]. Inflammatory cytokines (e.g., TNF-α, IL-1β and IL-10), chemokines (macrophage inflammatory proteins (MIP)-1α and monocyte chemotactic protein (MCP)-1) and TNFR-1 and TNFR-2 are widely used to evaluate the neuroinflammatory response in the presence of AD [8].

As demonstrated in some articles here reviewed, LPS injection up-regulated the release of TNF-α, IL-1β [46,47,52,53,54], MIP-1α [52] and MCP-1 [54] and down-regulated IL-10 expression [54] in the brain. Curcumin, TD, hesperetin and Se-Ps were efficacious in returning LPS-induced release of pro-inflammatory cytokines TNF-α and IL-1β to physiological levels [46,47,52,53,54]. In particular, the beneficial effects of curcumin in LPS-injected mice were mediated by TNFR-2 signaling [46], which is involved in the promotion of anti-inflammatory pathways in microglia [125] and protects against excitotoxity in neuropathological conditions [126]. Furthermore, in line with TD supplementation results on microglial activation, a dairy product fermented with *Penicillium candidum* suppressed Aβ accumulation and microglia overactivation in an AD mouse model (5xFAD) [127]. The decreased microglial overactivation due to TD could also prevent LTP suppression induced by LPS through the reduced expression of inflammatory cytokines, such as IL-1β and TNF-α [128,129]. Interestingly, curcumin was also able to attenuate LTP deficiency induced by LPS administration in rats [47]. 

As for pro-inflammatory chemokines, TD supplementation decreased the MIP-1α levels boosted by LPS injection [52], and Se-Ps were able to protect from the LPS-induced increase of MCP-1 [54]. Moreover, Se-Ps prevented the LPS-induced reduction of the anti-inflammatory cytokine IL-10 [54].

Similar beneficial effects of supplementation with plasmalogens, lycopene, hes-peretin, TD and selenium compounds on neuroinflammatory parameters have been also observed in other rat and mouse AD models [52,91,97,98,99,107,114,115].

NF-κB is well-recognized for its role in mediating both acute and chronic inflammatory responses, since it regulates the transcription of genes encoding cytokines, chemokines, pro-inflammatory enzymes and transcription factors, adhesion molecules and other factors that modulate the neuronal survival [130]. In mammals, NF-κB is composed by a family of five transcription factors: NF-κB1 (p105/p50), NF-κB2 (p100/p52), RelA (p65), RelB and c-Rel, which share sequence similarity over a 300-amino-acid region referred to as the Rel homology domain [131,132]. The inactive NF-κB resides ubiquitously in the cytoplasm of almost all cell types where it is linked to its inhibitor IκB. The activated NF-κB translocates from the cytoplasm to the nucleus, and the NF-κB-dimer can bind to the κB site of chromosomes to induce the transcription of NF-κB targeted genes. Canonical NF-κB-pathway activation may be mediated through a variety of cell-surface receptors, including TLR4, and in response to pro-inflammatory mediators, such as LPS. Depending on the cell type and the NF-κB subunits, activation of NF-κB pathway can have a dual role—acting either in neuroprotection or neurodegeneration [133]. Different studies on the transactivation of p65/p50 dimers showed the expression of proapoptotic genes, which cause neuronal death [134]. NF-κB signaling and p65/p50 dimer activation are elicited by LPS, Aβ_1–42_ and pro-inflammatory cytokines [135,136]. Both LPS and Aβ activate astrocytes and microglia, thereby, triggering the NF-κB signaling pathway and resulting in the up-regulated expression of pro-inflammatory cytokines (Figure 1). The exacerbated expression of these cytokines leads to a neuronal damage, which, in turn, causes neurodegeneration and the accumulation of Aβ plaques [137,138]. LPS injections significantly increase the brain expression of NF-κB and phosphorylated(p)-NF-κB [49,51,53], p-IκB [49,51], p65 and p-p65 levels [46,49]. Chicoric acid, lycopene and hesperetin were able to reduce NF-κB translocation and phosphorylation [49,51,53] and IκB phosphorylation [49,51], while curcumin and chicoric acid decreased p65 and p-p65 levels [46,49]. In particular, chicoric acid not only inhibited the NF-κB pathway but also the cytoplasmic signaling of mitogen-activated Protein kinase (MAPK) pathways in vivo, and it was able to suppress inflammation in vitro by inactivating MAPK/PI3K/Akt/NF-κB pathways in LPS-activated BV2 microglial cells [49]. p38 MAPK is an essential regulator of Aβ neurotoxicity and activates the NF-κB pathway eliciting the inflammatory response together with consequent synaptic plasticity impairment and excitotoxicity. On the contrary, inactivating p38 MAPK promotes anti-inflammatory and anti-apoptotic activities and preserves cognition [139]. Moreover, in addition to NF-κB, lycopene supplementation balanced the MAPKs and the erythroid 2 (NFE2)-related factor 2 (Nrf2) pathways, which could be the underlying mechanism involved in the effects of this nutraceutical [51]. In fact, together with the aforementioned MAPK and NF-κB mechanisms, the Keap1/Nrf2 pathway modulates inflammatory and oxidative responses, since, when oxidative stress increases, Nrf2 dissociates from Keap1, translocates to the nucleus and binds to antioxidant response element regulating the expression of antioxidants [140]. Moreover, dietary supplementation with chicoric acid or hesperetin counteracted the LPS-stimulated increase in TLR4 expression in mice brains [49,53]. 

Aβ aggregates engage with cellular receptors, such as TLRs and receptors for advanced glycoxidation end-products (RAGE), on both reactive astrocytes and microglia, thus, inducing the release of nitric oxide (NO) and reactive oxygen species (ROS), which also contribute to neuronal death [141,142,143,144]. LPS injections boost oxidative stress in the brain, increasing the levels of NO synthase (NOS) and the inducible isoform iNOS, while dietary supplementation with curcumin [46], Antarctic krill oil [48] or chicoric acid [49] effectively counteracted this effect. In accordance with these results, it has been previously reported that EPA and DHA can manage inflammation and oxidative stress by diminishing NF-κB activity, which, in turn, down-regulates the expression of iNOS and COX-2 genes [145,146].

Among ROS, particularly dihydroethidium (DHE) and malondialdehyde (MDA, a compound used as an oxidative stress marker), increased significantly in the brain of mice subjected to LPS injection, while diet supplementation with Antarctic krill oil [48], hesperetin [53] or Se-Ps [54] reverted this effect reducing oxidative stress. These results in LPS-injected rodents agree with the reduction of oxidative stress following supplementation with krill oil, hesperetin or organoselenium in other rat and mouse AD models [84,107,108,113]. In particular, hesperetin was able to regulate lactoperoxidase (LPO), ROS, Nrf-2 and Heme oxygenase-1 (HO-1). Furthermore, hesperetin rescued LPS-induced neuronal apoptosis by decreasing the expression of phosphorylated-c-Jun N-terminal kinases (p-JNK), B-cell lymphoma 2 (Bcl-2)-associated X protein (Bax) and caspase-3 protein and by increasing the Bcl-2 level. JNK is a stress kinase related to apoptotic processes and triggered by neuroinflammation and oxidative stress [147]. Bcl-2 and Bax are respectively anti- and pro-apoptotic protein markers, which regulate apoptosis at mitochondrial level [148]. Lastly, caspase-3 is a principal effector in apoptotic cascades resulting in neurodegeneration [149].

Finally, in evaluating brain oxidation, it could be useful to consider not only NOS and ROS markers but even antioxidants, such as glutathione (GSH), superoxide dismutase (SOD) and catalase (CAT), which are reduced in AD [150]. LPS injection lowered the brain levels of GSH, SOD and CAT antioxidants; however, dietary supplementation with lycopene enhanced GSH, SOD and CAT levels [51], and Se-Ps administration increased SOD and CAT [54]. In support of these findings, studies reported that selenium deficiency results in enhanced neuroinflammation and oxidative stress together with reduced antioxidant activities and decreased phagocytosis by macrophages in mice [151], while selenium supplementation can counteract apoptosis, inflammation and oxidative stress in aged rats with scopolamine-induced dementia [152].

## 4. Discussion

In the last decade, the interest in studying the overactivation of the immune system, which exacerbates AD by boosting neuroinflammation, has progressively increased [6]. In fact, growing evidence supports that AD pathogenesis is not only restricted to the neuronal level but also involves the immune system and that its dysregulated response to systemic inflammation has a fundamental role in neurodegeneration [8,11]. 

LPS injections are widely used to model systemic infection and elicit the activation of the immune system and the consequent cognitive and behavioral impairment in animals resembling the enhanced neuroinflammation of AD [33,34]. Lifestyle factors (e.g., nutrition, social activity, physical activity and leisure activity) could affect dementia susceptibility [153,154] because they affect brain and cognitive reserves and, consequently, overall cognitive functions in later life [155,156,157,158]. Many studies highlighted how healthy diet or dietary supplementation reduce the risk of AD or modulate the pathophysiology [79,159,160,161].

In this context, the present review was aimed at evaluating the impact of different nutrients and dietary supplements on the increase of neuroinflammation and the consequent cognitive decline in AD-like animal models of LPS administration. To date, the literature on this topic is still scarce. In fact, we found only nine articles published in the last 10 years describing the effects of dietary compounds concomitantly on cognitive function and neuroinflammatory and/or oxidative stress correlates after LPS administration. Moreover, all the selected articles addressed the effects of a single different dietary compound (except for curcumin); therefore, it is difficult to compare the effects among studies. Anyway, the two studies on curcumin agreed on the beneficial preventive effects of this compound on both cognitive decline and neuroinflammation induced by LPS [46,47]. Most of the bioactive compounds considered in the present review (i.e., curcumin, chicoric acid, lycopene, hesperidin and Se-Ps) are derived from plants. The remaining ones (Antarctic krill oil, plasmalogens and TD) have animal origins. All articles used chronic dietary supplementation (ranging from a minimum of 4 days, for curcumin, to a maximum of 3 months, for plasmalogens) in rodents. Four studies provided dietary supplementation (with curcumin, plasmalogens and TD) only before the LPS immunotoxic insult to address the preventive role of diet against AD neuropathology. In the remaining studies, dietary supplementation was initiated before LPS administration and continued during it, thus, exploiting a more prolonged nutraceutical action. 

To obtain systemic inflammation, single or repeated i.p. injections of LPS were performed in all studies, except for the one focusing on the neuroprotective role of TD in which LPS was only once intracerebroventricularly injected to induce inflammation directly in the brain. In mice, LPS was delivered with dosages of 250–300 μg/kg/day, while in the only study on rats, the LPS dosage f was 1 mg/kg in a single i.p. injection.

When dietary interventions with nutrients are administered to patients, off-target effects should be considered, exactly as for pharmacological treatments. These effects may be described as the uncontrolled biological activity of a molecule due to the modulation of other non-specific targets, which may provoke potential adverse effects, which can overwhelm the beneficial properties of the nutrient itself [162]. Therefore, it seems mandatory to explore the potential off-target effects even for nutraceuticals and adhere to the recommended dosages (which are based on extensive international scientific research) in order to build an efficacious treatment. In all studies revised, the researchers did not specify the presence or absence of any off-target or side/adverse effect related to the supplemented nutrients, except for one study in which it was reported that the chicoric acid supplementation showed no adverse effects on mice [49].

Despite the limitations due to the heterogeneity of the selected studies and the lack of off-target details, the studies reported the beneficial effects on the parameters of interest for this review (cognition, neuroinflammation and redox status), and many of them even investigated additional brain parameters (e.g., spine density, LTP, neurogenesis and apoptosis as discussed in detail above). Dietary supplementation appears to be able to modulate the overactivation of the immune system and, thus, to reduce the expression of inflammatory and oxidation markers through the inhibition of different cell-signaling processes—most importantly, the NF-κB pathway, which is mainly involved in LPS-induced neuroinflammation. This process results in reduced neuronal death and promotes neuronal survival together with increased resilience of the cognitive functions.

Taken together, these findings support the beneficial effects of a balanced and varied diet in contrasting neuroinflammatory insults and, thus, preventing conditions that favor AD. Furthermore, the study of individual nutrients lays the foundation to routinely integrate diet in clinical practice in a targeted and personalized manner, especially in those conditions in which specific nutritional or metabolic deficiencies more frequently occur. Indeed, many nutrients investigated in the present review—such as omega-3 fatty acids and plasmalogens—have shown reduced levels in elderly subjects and AD patients [89,163].

## 5. Conclusions

Pharmacological AD treatment today is focused on intervening in cognitive impairment and dysfunction of global activities through the use of cholinesterase inhibitors (donepezil, rivastigmine and galantamine), NMDA receptor modulators (memantine) and, most recently, immunotherapy (through the anti-Aβ monoclonal antibodies aducanumab and lecanemab). Currently, pharmacological approaches appear to be able to only temporarily relieve the symptomatology, and immunotherapeutic approaches show modest effects on cognitive decline, although the current literature is still controversial [164,165,166,167]. In this view, other interventional approaches are being sought. Dietary intervention is one of the most promising modifiable lifestyle factors in the prevention of AD due to its safety and low costs. The aim of the present review was to revise articles in which dietary supplementation was used as neuroprotective agent against the effects of LPS administration used in AD-like rodent models of enhanced neuroinflammation. Even though the selected studies presented heterogeneous compounds and methodological approaches, there is a general agreement on the protective effects that dietary supplementation may exert on cognitive decline, neuroinflammation and oxidative stress in mice and rats.

In conclusion, dietary interventions may represent a strong resource to fight AD neuropathology. Considering the limited number of papers currently found, further investigations are needed to explore the mechanisms of action of dietary compounds on neuroinflammatory and oxidative stress featuring AD neuropathology (Box 1).

Box 1 **Summary of key findings and future aspects to be explored.**

Concluding remarks:

- Dietary interventions appear to be efficacious in counteracting LPS-induced cognitive impairment in rodents.
- The cognitive benefits are accompanied by a reduction of neuroinflammation and oxidative stress.
- Dietary interventions could be a strong resource in fighting AD due to their safety and low costs.

Open questions:

- How difficult and expensive would it be to diagnose nutrient deficiencies in single MCI or AD patients in the first stages of the pathology?
- How can we integrate dietary interventions with MCI or AD pharmacological therapies in order to be sustainable and effortless for patients and caregivers and, thus, reduce dropout?
- Dealing with a multitude of nutrients, what strategies could be used to improve their selection and manage off-target and potential adverse effects?

## Figures and Tables

**Table 1:** Summary of the methodological approaches of the articles analyzed in the literature review.

Article	Animal Age and Strain	Kind of Dietary Supplementation	LPS Administration Procedure	Cognitive Assessment Methods	Biological Correlates
Kawamoto et al., 2013 [46]	Male mice 12- to 14-week-old TNFR1-TNFR2 double knockout and C57BL/6J	Curcumin 50 mg/kg i.p. injection For 4 days	250 μg/kg Intraperitoneal (i.p.) injection (single) 2 h after the last vehicle/curcumin administration	Morris Water Maze Fear Conditioning test After supplementation and LPS administration	Immunoblot analysis: NR1 and RelA; EAAT2 and pSer897-NR1 hnRNP C1/C2; TNFR1 and TNFR2; EAAT3; GFAP; GluR1; pSer845GluR1; NOS. ELISA: TNF-α; IL-1β.
Hajipour et al., 2023 [47]	Male rats 180–200 g; adult rats, age not specified Wistar	Curcumin 50 mg/kg gavage (suspended in a 1% methylcellulose) For 14 days	1 mg/kg, i.p. injection (single) 1 h after the last vehicle/curcumin administration	Step-through Passive Avoidance test 24 h after LPS administration	Histological analysis: Hematoxylin-eosin ELISA: TNF-α; IL-1β Electrophysiology: Long-term Potentiation (LTP) recordings
Choi et al., 2017 [48]	Male mice 8- to 10-week-old ICR	Antarctic krill oil 80 mg/kg supplemented rodent chow (5% krill oil)For 4 weeks	250 μg/kg/day i.p. injection During the last 7 days of supplementation	Morris Water Maze Step-through Passive Avoidance test After supplementation and LPS administration	Histological analysis: GFAP; IBA-1; iNOS; COX-2; DHE. Western blot: APP; IBA-1; iNOS; BACE1; COX-2; GFAP. ELISA: Aβ_1–42_ Oxidative Stress Assay Assay of β-Secretase Activities
Liu et al., 2017 [49]	Male mice 3-month-old C57BL/6J	Chicoric acid 0.05% in drinking water For 45 days	0.25 mg/kg/day i.p. injection For 9 days during supplementation	Spontaneous alternation Y-maze test Morris Water Maze During supplementation and LPS administration (starting 4 h after the first LPS injection)	Histological analysis: GFAP; IBA-1; Aβ_1–42_; Hematoxylin-eosin; Thioflavin S; NeuN; BrdU; DAPI. ELISA: Aβ_1–42_; ACh; AChE; ChAT. Western blot: COX-2; GAPDH; lamin B; iNOS; NF-kB; p-p44/42 MAPK (ERK1/2); p44/42MAPK (ERK1/2); p-SAPK/JNK (Thr183/Tyr185); SAPK/JNK; p-p38MAPK; p38MAPK; IkB; p-IkB; APP; p-NF-kB; BACE1; p50; p-65; p-AKT; AKT.
Hossain et al., 2018 [50]	Male mice 7-month-old C57BL/6J	Plasmalogens 0.1 mg/mL and 10 mg/mL in normal drinking water replaced every 2 days For 3 months	250 μg/kg/day i.p. injection For 7 days starting after supplementation	Morris Water Maze After supplementation and LPS administration	Histological analysis: GFAP; IBA-1; Aβ; DAPI.
Wang et al., 2018 [51]	Male mice 3-month-old C57BL/6J	Lycopene 0.03%, *w*/*w* mixed with standard diet For 5 weeks	0.25 mg/kg/day i.p. injection For the last 9 days of supplementation	Spontaneous alternation Y-maze test Morris Water Maze 4 h after LPS administration	Histological analysis: IBA-1; Aβ_1–42_; Hematoxylin-eosin. ELISA: Aβ_1–42_; levels of GSH; activity of CAT and SOD. Western blot: COX-2; lamin B; HO-1; NQO-1; Keap1; and Nrf2; APP; BACE1; NF-κB; IκB; p-IκBα; p-p44/42 MAPK (ERK1/2); p44/42 MAPK (ERK1/2); p-SAPK/JNK (Thr183/Tyr185); SAPK/JNK (9252); p-p38 MAPK; p38 MAPK; p-AKT; AKT.
Ano et al., 2019 [52]	Male mice 6-week-old ICR	Tryptophan-related dipeptides in fermented dairy products 0, 3 or 30 mg/kg/day dissolved in distilled water For 8 days	5 μg Intracerebroventricular (i.c.v.) injection (single) 30 min after the last supplementation	Spontaneous alternation Y-maze test 3 days after LPS administration Novel Object Recognition Test 4–5 days after LPS administration	Histological analysis: GolgiStain for spines count of the CA1. -ELISA: TNF-α; IL-1β; macrophage inflammatory protein 1α (MIP-1α).
Muhammad et al., 2019 [53]	Male mice 7- to 8-week-old C57BL/6N	Hesperetin 50 mg/kg/day gavage (suspended in water) For 5 weeks	250 μg/kg/day i.p. injection For the last 2 weeks of supplementation (a total of 7 doses administered on an alternate day)	Morris Water Maze Spontaneous alternation Y-maze test During the last week of supplementation and LPS administration (1 h after both administrations)	Histological analysis: GFAP; p-NF-κB; TNF-α; p-CREB; DAPI and Nissl’s Staining for neuronal cell loss. Western blot: TLR4; Iba-1; GFAP; p-NF-κB; TNF-α; HO1; IL-1β; Nrf2; p-JNK; Bax; PSD-95; Bcl2; Syntaxin; p-CREB; Cl-Caspase-3. In Vivo ROS and LPO Assays.
Wu et al., 2022 [54]	Male mice 6-week-old ICR	Selenium Peptides 10 or 30 mg/kg/day BW gavage (suspended in distilled water) For 28 days	300 μg/kg/day i.p. injection For 9 days (from day 21 to 28 of supplementation)	Step-down Passive Avoidance test After supplementation and LPS administration	ELISA: MCP-1; IL-1β; TNF-α; IL-10; MDA; CAT; SOD.

## Data Availability

Not applicable.
